# Architectural Narrative Shapes Brain Activities Underlying Approach-Avoidance Response: A Case Study of the Stadium

**DOI:** 10.3389/fnins.2022.858888

**Published:** 2022-05-13

**Authors:** Weixia Zhang, Hongyang Wei, Xiaowen Chen, Yuyang Hou, Yujie Zhang, Qian Huang

**Affiliations:** ^1^Department of Physical Education, Northwestern Polytechnical University, Xi’an, China; ^2^Graduate Department, Xi’an Physical Education University, Xi’an, China; ^3^Graduate Department, Shanghai University of Sports, Shanghai, China; ^4^School of Sports Training, Xi’an Physical Education University, Xi’an, China

**Keywords:** architectural narrative, building, stadium, continuity editing, late positive potential, emotion

## Abstract

Each great architecture tells a story to make its space meaningful. What the stadium tells matters how the individual interacts with it. The potent influence of narrative in shaping our cognitive processing has been revealed and widely used. This influence, however, has not been the focus of researchers in stadium operations. The present study aimed at investigating the influence of the stadium narrative on approach-avoidance responses and the corresponding neural correlates. Participants were presented with a sequence of pictures expressing a story congruent or incongruent with the general profile of the stadium, and were required to make an enter or exit response. Results showed larger amplitudes of N400 for incongruent trials than congruent trials at the end of the narrative, indicating the feasibility of continuity editing procedure for the study of narratives. Moreover, larger amplitudes of LPP were observed in response to the stadium preceded by congruent trials than incongruent trials. This effect was more pronounced in the left than right frontal sites. The LPP suggested that a congruent narrative imparted the stadium approaching affective features, and induced approaching responses, which was consistent with the behavioral and correlational results. Our findings suggested that changes in narrative were sufficient to shape the approach-avoidance responses and the underlying neural correlates. Implications for stadium management and buildings are provided.

## Introduction

We live and work in buildings made of concrete and steels, and the things that connect us between such buildings are called stories (Hinton, 2014). By telling stories, the building itself becomes a meaningful space rather than a mere physical object, this process is known as the narrative (DiMascio and Maver, 2014; Jamieson, 2017). Architectural narrative prioritizes human experience in buildings and concerns about what happens in space (Tseng, 2015). Architectural narrative not only expresses social and cultural messages of the semantic meanings of buildings and space, but also contributes to the construction of meaning *per se* (Psarra, 2009). A narrative thus becoming an essential element in the design of architecture (Tissink, 2016). A strong architectural narrative may add interests for its users and stamp their experiences with narrative feature (Chen and Kalay, 2008). As an architecture, the stadium is no longer portrayed as a silent physical matter, but actually as an epitome of culture in the city (Sheard, 1985) and an actor that explained its own identity. Therefore, what stories the stadium tells or what can be read from the stadium counts a lot from the perspective of architectural narrative.

The narrative is not only a medium whereby which we interact with the environment; studies have shown that it is a potent factor that shapes our processing of information (Wheeler et al., 1999; Green and Brock, 2000; Shiller, 2017; Piotrowski et al., 2019). Behavioral studies showed that narratives could induce our attitudes to be more congruent with that expressed in a narrative (Wheeler et al., 1999; Green and Brock, 2000), and the influence of a narrative can even override statistical information (Betsch et al., 2011). Moreover, by manipulating the temporal structure of stories, researchers collected fMRI data when presenting participants with either an intact temporal sequence of the story, or with the same event in scrambled order. The results showed that the temporally scrambled events evoked weaker schematic representations in mPFC (Baldassano et al., 2018). The effect of a narrative on neural responses persists even after the disappearance of stimuli, as study showed functional connectivity changed after listening to narratives representing different emotional contents (Borchardt et al., 2015). This persisting effect was also obviously revealed by using EEG (Borchardt et al., 2018). These studies showed the feasibility of using neuroscience technique to investigate the effect of narrative, as well as the potential neural correlates related to the influence of narrative. Although the narrative is commonly related to literature and plays, it is also widely accepted in architectures (Jo and Lee, 2007; Wakefield, 2015). It is reasonable to postulate that the influence of narrative also exists in architectures.

The powerful influence of a narrative has been widely used, such as in communicating effectively with the general about the policies of government (Piotrowski et al., 2019), and in addressing the economic fluctuations (Shiller, 2017). However, the potent effect of a narrative seems to be neglected by researchers in the stadium management, in which exploring the determinants of stadium attendance is of pivotal importance (Schreyer and Ansari, 2021), as getting revenue from ticket sales is an important part of financial resources (Deloitte, 2021). Related to this issue are studies concerned about how built environments affect human behavior. Emotion may be a core node in this relationship as shown by the structural equation modeling analysis (Ryu and Jang, 2007). Naturally, environmental factors were the mostly studied variable related to stadium attendance (Uhrich and Koenigstorfer, 2009; Chen et al., 2013; Yoshida et al., 2021). Consistent with studies in other buildings (Ryu and Jang, 2007), findings showed that the environmental stimuli of the stadium affected an individual’s behavioral response *via* emotional processing (Uhrich and Koenigstorfer, 2009; Chen et al., 2013; Yoshida et al., 2021). Specifically, the more satisfied people with the environmental stimuli (Chen et al., 2013; Yoshida et al., 2021) or more positive emotion they experienced (Uhrich and Koenigstorfer, 2009), the more likely they attended the stadium. These studies highlighted the role of emotion in responding to the environmental stimulus.

The relationship between environmental stimulus and people’s responses could be supported by the S (stimuli)–O (organism)–R (response) model, which assumes that stimuli in the environment affect an individual’s emotional states and ultimately result in approach or avoidance responses (Mehrabian and Russell, 1974). Approach is positive, reflecting the desire to stay in the environment while avoidance is negative, indicating the withdrawal behavior (Mehrabian and Russell, 1974). From the perspective of environmental psychology (Mehrabian, 1976), the stadium narrative can be regarded as an environmental stimulus and the narrative itself is a potent medium for emotion-eliciting (Westermann et al., 1996). Thus, it is likely that the stadium narrative would potentially influence approach-avoidance response toward the stadium by internal emotion processing of the individual. However, no study has been conducted to reveal the causal link between stadium narrative and approach-avoidance response, and the mechanisms underlying this link also remains unknown. The employment of techniques of neuroscience provides a method to reveal the internal psychological processing of organism and helps to advance our understanding of the mechanisms underlying a certain behavior (Churchland and Sejnowski, 1988; Bell and Cuevas, 2012). Among all the techniques, electroencephalography (EEG) records brain electrical activities at a millisecond in an non-invasive manner and is relatively low-costing, and has been used in engineering (Hou et al., 2021; Liu et al., 2022).

Therefore, the present study aimed at investigating the influence of the stadium narrative on approach-avoidance response and its neural correlates by using EEG. First, a new experimental procedure, which was called “continuity editing procedure” here, was introduced to enable the stadium to tell a story based on filmmaking theory. Continuity editing is a technique widely used in filmmaking. In continuity editing, many camera shots are used to create a continuous sequence of events that comprise a narrative (Magliano and Zacks, 2011). Here, participants were presented with sequences of pictures forming two different conditions. Each picture represents a unique scene in the sequence. The sequences are presented progressively from the spatial perspective. As the narrative goes, participants would feel that they are getting closer and closer to the stadium. The essence of the difference between congruent and incongruent condition is the continuity of events. In one condition, pictures are sequenced to express the theme that “This is a stadium with wonderful games.” In another condition, the theme is “This is a stadium in which people do nothing related to sports but working or studying.” The distinction in the two conditions is achieved by using either sports-related or sports-unrelated pictures at the end of the narrative. Because events that have happened or have been happening in it have naturally become a part of the source of narratives (Tseng, 2015), and the stadiums are often used for exhibiting sports games, the narrative contents in the two conditions are either congruent or incongruent with what are generally happening in the stadium. The two conditions were called “congruent condition” and “incongruent condition” hereafter, respectively. In the congruent condition, events happened in the stadium could be naturally continued. In the incongruent condition, events happened in the stadium were somewhat abrupt, or were less continued than in the congruent condition. A similar experimental design could be seen in other studies related to the narrative by using continuity editing (Speer et al., 2007; Magliano and Zacks, 2011).

Second, the N400 component was used to verify the feasibility of our experimental procedure. The N400 is a negative component peaking at approximately 400 ms after stimuli onset, is a marker of semantic processing (Berkum et al., 1999; Kutas and Federmeier, 2011), and is sensitive to semantic expectancy (Kutas and Hillyard, 1980). If larger N400 amplitude was induced in incongruent trials than that of congruent trials, then this indicated that pictures on the marker point were more expected in congruent trials than incongruent trials. On the other hand, this suggested that the presented sequences could convey information in a way similar to language, reflecting that the experimental procedure is effective at enabling the stadium to speak.

Third, the LPP (late positive potential) would be focused in data analysis. The LPP is a broadly distributed positivity with the average latency starting at approximately 300 ms and lasting for several milliseconds after stimulus onset (Cuthbert et al., 2000). The LPP is thought to index the emotional processing as larger amplitudes of LPP were found in response to affective pictures than non-affective neutral ones (Cuthbert et al., 2000; Schupp et al., 2000; Olofsson et al., 2008; Hajcak et al., 2010). Moreover, studies revealed larger LPP over the left frontal cortex in response to approach-related stimuli, such as appetitive pictures (Gable and Harmon-Jones, 2010), and concepts that were rated as good like welfare (Cunningham et al., 2005), while avoidance-related stimuli elicited larger LPP amplitudes in the right frontal (van de Laar et al., 2004; Cunningham et al., 2005). The frontal lateralization of the LPP is consistent with findings that showed the asymmetry in frontal cortex in emotional processing [for a review, see Harmon-Jones et al. (2010)]. Therefore, differences in the amplitudes of LPP between two conditions could be explained by emotional processing or the processing of approach-avoidance stimuli if the LPP effect has a frontal lateralization. Considering that environmental stimuli influenced people’s emotional connection with the stadium at the behavioral level (Uhrich and Koenigstorfer, 2009; Chen et al., 2013; Yoshida et al., 2021), it is assumed that significant differences in LPP would be observed in the two conditions. It is further hypothesized that frontal lateralization of LPP would be different between the two conditions, if significant behavioral responses exist.

## Materials and Methods

### Participants

Sample size was estimated using G*Power (version 3.1) (Faul et al., 2007). Twenty-six participants would be required to detect statistically significant *F*-test difference with a power of 0.85 at a significance level of *p* = 0.05. Therefore, thirty university students were recruited as paid volunteers, two of which were removed for further analysis due to abnormal recording. In the end, twenty-eight participants were included in our experiment (*Mage* = 24.07 years, *SD* = 1.75 years; 15 females). All participants were right-handed, with no history of neurological or psychiatric diseases. All reported having normal or corrected-to-normal vision. None had received any extracurricular training in architecture design and sports. Before the experiment, all participants signed a written consent form.

### Stimuli

Four kinds of pictures were used in the presentation of sequences: (1) the outer appearances of the stadium; (2) the inside of the stadium; (3) pictures related to the stadium such as sports games; (4) pictures unrelated to the stadium such as working or studying. Studying- or working-related pictures rather than random pictures like animals or plants were chosen to avoid generating potential confounding variables. As all contents of the pictures were confined to the scope of human activities, and the essential difference of the pictures between the two conditions was level of activities, the congruent condition was active while the incongruent was sedentary. This selection would make the concepts associated with the two conditions comparable.

First, 960 pictures were downloaded and selected from the Internet, with 240 pictures for each kind. Pictures with resolution above 600 × 800 pixels were selected. After the selection, some of the pictures were further edited using Photoshop software to remove information that interferes with the expression of the theme, such as crowds outside the stadium. Then, pictures were sequenced to form two conditions. In the congruent condition, pictures were arranged with the sequence “outer appearance of the stadium in distance-outer appearance of the stadium-the inside stadium-sports related scene-outer appearance of the stadium in distance” to tell that “This is a stadium with wonderful games.” In the incongruent condition, pictures were arranged with the same sequence except that the fourth pictures were replaced by pictures unrelated to sports to tell that “This is a stadium in which people do nothing related to sports but working or studying.” Finally, 162 sequences were selected as the final experimental stimuli. Other sequences were excluded due to inappropriate arrangement of the pictures. The inappropriate arrangement of the pictures refers to the sequences that cannot bring a sense of congruence for the congruent trials or a sense of incongruence for the incongruent trials. For example, the combination of a football field with basketball games. For the final 162 sequences, half were congruent while the other half were incongruent.

### Procedure and Design

**FIGURE 1:**
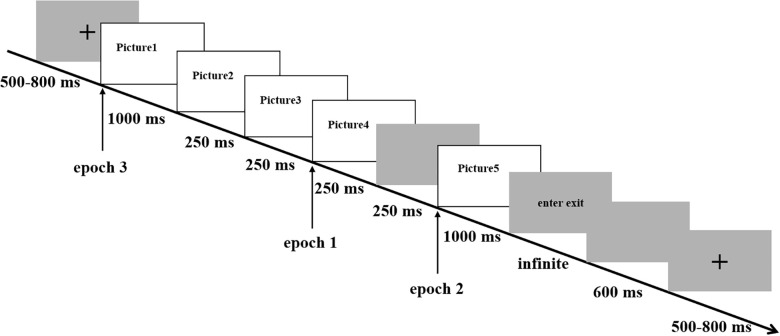
Flow of experimental procedure and design.

Each trial started with a black fixation in the middle of the screen with a light gray background for 500–800 ms. Then, the first picture was presented for 1,000 ms, followed by the second, the third, and the fourth pictures, each presented for 250 ms. To generate a sense of narrative, we presented the pictures in the following sequence: stadium in the distance, stadium in a nearer site, the interior of a stadium, and the things happening in a stadium accordingly. After the presentation of the preceding four pictures was a 250 ms blank screen. At the end, the stadium reappeared on the screen for 1,000 ms. Following was the response interface, lasting for infinite time until participants made a response. Such a design would avoid any contamination from artifacts associated with the actions of button-pressing. During the experiment, all stimuli were presented on a 20-inch Dell computer monitor (1,440 × 900 pixels, 60 Hz), situated approximately 100 cm from the participant. See Figure 1 for more details.

Participants were instructed to express their approach-avoidance attitude toward the stadium by pressing either enter or exit buttons. The association between the hand side (left or right) and the response (enter or exit) was counterbalanced across the participants. The enter and exit responses represent expression of approach and avoidance decisions, respectively (Vartanian et al., 2013). Before the formal experiment, six practice trials were given to familiarize the participants with the stimuli and procedure.

As pictures in congruent and incongruent conditions are different in the study, the physical stimulus confounds are unavoidable (Luck, 2005). Thus, the possible significant differences cannot be totally attributed to our manipulation. To address this issue, we would present the stadium two times and analyze data after subtracting ERPs induced by the reappearance of the stadium from ERPs induced by the stadium that appeared for the first time. If the results have the same pattern, this indicates that the significant differences in the two conditions are not contaminated by physical differences.

### Electroencephalography Recording and Preprocessing

The EEG was recorded from 64 Ag/AgCl electrodes organized according to the international 10/20 system. The electrode in front of the Fz served as ground. Four additional electrodes were placed at the left eye supra- and infraorbitally and at the outer canthi of both eyes to record vertical and horizontal electrooculograms (EOGs), respectively. Impedances of all electrodes were kept below 10 kΩ during recording. The sampling rate was 1,000 Hz, with a band-pass filter of 0.05–100 Hz.

The preprocessing was conducted using Brainstorm (Tadel et al., 2011). EEG was resampled at 512 Hz and re-referenced to the average of bipolar mastoid. A band-pass filter of 1–30 Hz was applied offline. Eye movements were corrected by using independent component analysis (ICA). Noisy segments were excluded manually. EEG epochs from −200 to 1,000 ms relative to the first, the fourth, and the fifth pictures’ onset were time-locked and baseline corrected (−200–0 ms). That is, there were three types of EEG epochs in the study (see Figure 1). The first epoch was based on the onset of the fourth picture in the sequence (end of the narrative); it was used for confirming the feasibility of the experimental procedure. The fourth picture was situated at the end of the narrative. Pictures preceding the fourth picture served as a context. Only after the context is established can we investigate whether or not the subsequent stimulus fits well with it. Therefore, similar to the classic study of N400, in which the stimulus was presented word by word and ERPs were extracted at the end of the sentence (Kutas and Hillyard, 1980), the present study displayed information picture by picture and analyzed the N400 effect for the fourth picture. The second epoch was based on the onset of the fifth picture in the sequence (reappearance of the stadium); it was used for exploring the influence of preceding narratives. The third epoch was based on the onset of the first picture in the sequence, and it was used for excluding the compounds of physical differences in different conditions.

### ERP Data Analysis

All ERP analyses were based on the mean amplitude values of each participant under each condition. Based on visual inspection and previous studies concerned about N400 (Kutas and Federmeier, 2011) and LPP (Gable and Harmon-Jones, 2013; Gable and Poole, 2014), the time window of 200–400 ms was used for statistical analysis for the fourth picture in sequence. For the first and fifth pictures in sequence, 500–800 ms were used for statistical analysis.

Repeated-measures ANOVAs were conducted for the midline (anterior: FPZ and FZ; central: FCZ, CZ, and CPZ; posterior: PZ, POZ, and OZ) and lateral electrodes (left anterior: AF3, F1, F3, and F5; right anterior: AF4, F2, F4, and F6; left central: FC1, FC3, FC5, C1, C3, and CP1; right central: FC2, FC4, FC6, C2, C4, and CP2; left posterior: P1, P3, and CB1; right posterior: P2, P4, and CB2) separately. For the midline electrodes, congruency (congruent and incongruent) and anteriority (anterior, central, and posterior) were considered as within-subjects factors. For the lateral electrodes, hemisphere (left and right) was added as an additional within-subjects factor, that is, a 2 (congruency: congruent and incongruent) × 3 anteriority (anterior, central, and posterior) × 2 (hemisphere: left and right) repeated-measures ANOVA for lateral analysis. The mean value of the electrodes in each region of interest was computed for analysis. The analysis of simple effects tests if there were obvious interactions and all pairwise comparisons were adjusted by Bonferroni correction. Only the significant effects containing the congruency (the main variable) were reported.

## Results

### Behavioral Results

**FIGURE 2:**
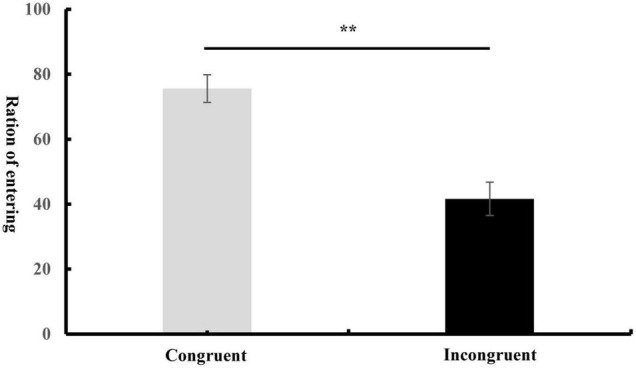
Values of “enter” ratio under each condition. The error bars refer to the standard errors.

A paired *t*-test was conducted based on the ratio of “enter” response for each participant under each condition. As shown in Figure 2, the results showed a significantly higher value of “enter” response [*t*_(27)_ = 4.31, *p* < 0.001, *d* = 1.51] for congruent condition (*M* = 75.61%, *SD* = 22.53%) than incongruent condition (*M* = 41.64%, *SD* = 27.36%). Although response time was collected, we would not analyze it given that the response was delayed in the present study.

### Electrophysiological Results and Correlational Analysis

#### Epoch1 (The Fourth Picture in the Sequence)

**FIGURE 3:**
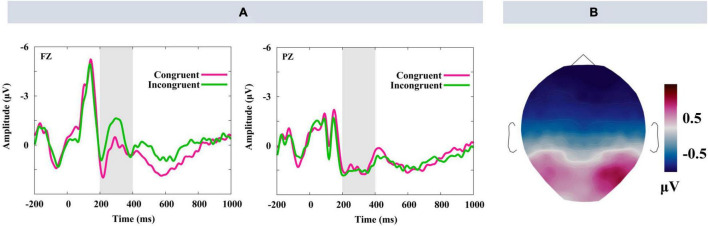
Grand mean ERP waveforms elicited by the fourth picture. **(A)** FZ and PZ. Gray-shaded areas indicate the time windows used for statistical analysis. **(B)** Displayed the Scalp distribution of the incongruent-minus-congruent difference waves in the time window of 200–400 ms.

Figure 3 shows the grand average waveforms elicited by the fourth picture in the sequence (A) and the scalp distribution of incongruent-minus-congruent difference waves (B). As can be seen, a larger N400 was induced by the incongruent than congruent trials. This N400 effect was globally distributed and maximal between 200 and 400 ms.

Statistical analysis of the N400 time window of 200–400 ms showed main effects of congruency on midline electrodes [*F*_(1,27)_ = 6.10, *p* = 0.02, η^2^ = 0.18] and lateral [*F*_(1,27)_ = 8.71, *p* = 0.01, η*^2^* = 0.24], as a larger N400 amplitude was elicited under the incongruent condition than under the congruent condition. Significant interactions were observed between anteriority and congruency on both midline [*F*_(1.33,35.87)_ = 17.25, *p* < 0.001, η*^2^* = 0.39] and lateral electrodes [*F*_(1.17,31.47)_ = 26.19, *p* < 0.001, η*^2^* = 0.49], as there were larger N400 in incongruent vs. congruent trials for the anterior electrodes [midline: *F*_(1,27)_ = 16.02, *p* < 0.001, η*^2^* = 0.37; lateral: *F*_(1,27)_ = 21.50, *p* < 0.001, η*^2^* = 0.44] and central electrodes [midline: *F*_(1,27)_ = 8.20, *p* = 0.01, η*^2^* = 0.23; lateral: *F*_(1,27)_ = 12.98, *p* = 0.001, η*^2^* = 0.33], but not for the posterior electrodes [midline: *F*_(1,27)_ = 3.23, *p* = 0.08; lateral: *F*_(1,27)_ = 3.36, *p* = 0.08].

#### Epoch2 (The Fifth Picture in the Sequence)

**FIGURE 4:**
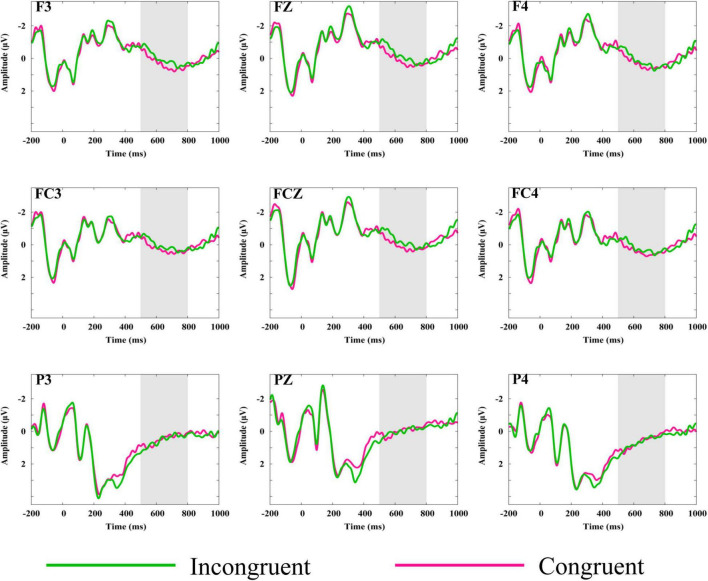
Grand mean ERP waveforms elicited by the reappearance of the stadium preceded by congruent or incongruent narratives. Gray-shaded areas indicate the time windows used for statistical analysis. F3, FZ, F4, FC3, FCZ, and FC4 showed larger amplitude of LPP for congruent trials than in congruent trials, while P3, PZ, and P4 showed no significant difference between the two conditions.

**FIGURE 5:**
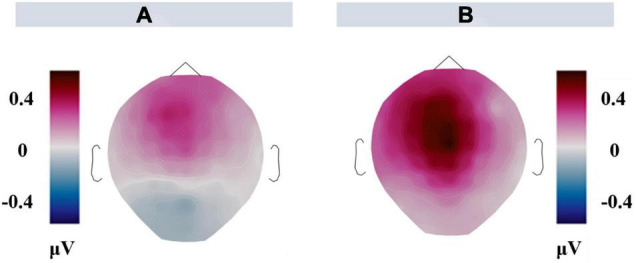
**(A)** Scalp distribution of the congruent-minus-incongruent difference waves in 500–800 ms. **(B)** Scalp distribution of the congruent-minus-incongruent difference waves in 500–800 ms derived from the values of fifth picture minus the first picture. That is, values in congruent trials (values of the fifth picture minus the first picture in congruent condition) minus values in incongruent trials (values of the fifth picture minus the first picture in incongruent condition).

Figure 4 shows the grand average waveforms elicited by stadiums (the fifth picture in the sequence) preceded by congruent and incongruent narrative. Figure 5 shows the scalp distribution of congruent-minus-incongruent difference waves (A). For the fifth picture in the sequence, a larger LPP was induced by the congruent than incongruent trials in the time window of 500–800 ms.

Analysis of the LPP in the time window of 500–800 ms revealed a significant main effect of congruency on midline [*F*_(1,27)_ = 4.28, *p* = 0.048, η*^2^* = 0.14], as congruent trials elicited a larger LPP than incongruent trials. Significant interactions between congruency and anteriority were observed on both midline [*F*_(1.19,32.25)_ = 5.47, *p* = 0.02, η*^2^* = 0.17] and lateral electrodes [*F*_(1.17,31.51)_ = 4.94, *p* = 0.03, η*^2^* = 0.16], as larger amplitudes of LPP were elicited by congruent trials than incongruent trials for the anterior electrodes [midline: *F*_(1,27)_ = 7.15, *p* = 0.01, η*^2^* = 0.21; lateral: *F*_(1,27)_ = 6.77, *p* = 0.02, η*^2^* = 0.20] and central electrodes [midline: *F*_(1,27)_ = 7.32, *p* = 0.01, η*^2^* = 0.21; lateral: *F*_(1,27)_ = 4.81, *p* = 0.04, η*^2^* = 0.15], but not for the posterior electrodes [midline: *F*_(1,27)_ = 0.95, *p* = 0.34; lateral: *F*_(1,27)_ = 0.59, *p* = 0.45].

#### Epoch2-Minus-Epoch3

**FIGURE 6:**
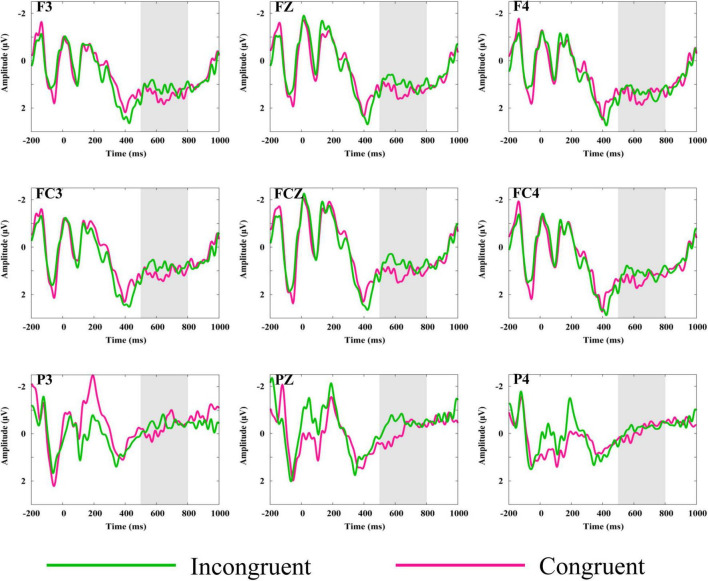
Grand mean ERP waveforms of the congruent-minus-incongruent difference waves derived from the values of fifth picture minus the first picture. Gray-shaded areas indicate the time windows used for statistical analysis. F3, FZ, F4, FC3, FCZ, and FC4 showed larger amplitude of LPP for congruent trials than in congruent trials, while P3, PZ, and P4 showed no significant difference between the two conditions.

To exclude the above LPP effect (congruent-minus-incongruent) was contaminated by physical differences in the congruent and incongruent conditions, the value of amplitudes elicited by the fifth picture minus the first picture was treated as a dependent variable. Figure 6 shows the grand average of difference waveforms elicited by the fifth picture minus the first picture in the sequence. Figure 5 shows the scalp distribution of congruent-minus-incongruent difference waves for epoch2-minus-epoch3 in the time window of 500–800 ms (B).

Analysis of the LPP in the time window of 500–800 ms showed significant main effects of congruency on midline [*F*_(1,27)_ = 10.05, *p* = 0.04, η*^2^* = 0.27] and lateral [*F*_(1,27)_ = 9.94, *p* = 0.004, η*^2^* = 0.27], as larger positivity was elicited under congruent condition than the incongruent condition. Moreover, interactions between anteriority and congruency on both midline [*F*_(2,54)_ = 3.93, *p* = 0.03, η*^2^* = 0.13] and lateral electrodes [*F*_(2,54)_ = 3.44, *p* = 0.04, η*^2^* = 0.11] were observed, owing to larger LPP in the congruent than incongruent trials for the anterior [midline: *F*_(1,27)_ = 6.79, *p* = 0.02, η*^2^* = 0.20; lateral: *F*_(1,27)_ = 6.82, *p* = 0.02, η*^2^* = 0.20] and central electrodes [midline: *F*_(1,27)_ = 17.47, *p* < 0.001, η*^2^* = 0.39; lateral: *F*_(1,27)_ = 16.69, *p* < 0.001, η*^2^* = 0.38], but not for the posterior electrodes [midline: *F*_(1,27)_ = 0.09, *p* = 0.77; lateral: *F*_(1,27)_ = 0.1, *p* = 0.92].

#### Laterality of the Frontal Late Positive Potential

Although the interactions between congruency and hemisphere was not significant in the above repeated-measures ANOVAs, there were theoretical motivations to examine the laterality of LPP (Maxwell et al., 2017). Because larger amplitudes of LPP in the left frontal hemisphere are related to approach motivation (Gable and Harmon-Jones, 2013; Gable and Poole, 2014), and our behavioral results showed a higher ration of “enter” under congruent than incongruent condition, we speculated that the difference caused by congruent-minus-incongruent trials should be more pronounced in the left frontal area than in the right frontal area. Therefore, similar to previous studies concerned about the laterality of LPP (Gable and Harmon-Jones, 2010; Gable and Harmon-Jones, 2013), we choose AF3, F1, F3, and F5 for the left lateral frontal electrodes and AF4, F2, F4, and F6 for the right lateral frontal electrodes. A paired *t*-test was conducted based on the mean difference value (congruent minus incongruent) of LPP in response to the fifth picture. The result showed a significant greater LPP effect in the left frontal than in the right frontal [*t*_(27)_ = 2.75, *p* = 0.01, *d* = 0.24]. The result remained the same after excluding the physical differences of the stadium in congruent and incongruent conditions [*t*_(27)_ = 3.29, *p* = 0.003, *d* = 0.25].

The correlation between congruent-minus-incongruent LPP amplitudes and behavioral response was conducted. The results showed significant positive correlation in anterior electrodes (*r* = 0.67, *p* < 0.001), central electrodes (*r* = 0.51, *p* = 0.01), left anterior electrodes (*r* = 0.65, *p* < 0.001), right anterior electrodes (*r* = 0.64, *p* < 0.001), left central electrodes (*r* = 0.45, *p* = 0.02), and right central electrodes (*r* = 0.50, *p* = 0.01). No obvious relationship was found in posterior electrodes (*r* = −0.29, *p* = 0.13), left posterior electrodes (*r* = −0.28, *p* = 0.15), and right posterior electrodes (*r* = −0.29, *p* = 0.14).

## Discussion

By using ERPs, the present study investigated the effects of the stadium narrative on the approach-avoidance response toward the stadium, and the corresponding neural correlates underlying such effects. The results showed larger amplitudes of N400 during the time window of 200–400 ms for incongruent trials than congruent trials at the end of the narrative, reflecting the feasibility of the experimental procedure. In the time window of 500–800 ms after the reappearance of the stadium, larger amplitudes of LPP were observed preceded by congruent trials than incongruent trials. The LPP effect was invulnerable to the physical differences in the two conditions and has a left frontal laterality. Our findings suggested that changes in the stadium narratives affected approach-avoidance responses and the corresponding neural correlates.

The first finding was that a more negative N400 was observed at the end of the narrative for incongruent trials than congruent trials. The N400 effect in the present study indicated that the stories in incongruent trials were less expected than stories in the congruent trials, as the amplitude of N400 reflected semantic expectation (Kutas and Hillyard, 1980). On the other hand, the N400 effect in the study suggests that the continuity editing procedure could convey information in a similar way to language, confirming the feasibility of continuity editing procedure for the study of architectural narrative. The finding of N400 suggested that a stadium was not simply a silent building, but also a lively actor that embodied its own identity and stories.

Another finding was that larger amplitudes of LPP were elicited for congruent trials compared with incongruent trials when the stadium reappeared. Given that affective pictures evoke larger amplitudes of LPP than non-affective or neutral pictures (Schupp et al., 2004; Pastor et al., 2008), the LPP effect in the present study indicated that the stadium narrative imparted the stadium the affective meanings. Specifically, compared with the condition in which the stadium was associated with non-sports events, the condition in which the stadium was associated with sports tends to embody more affective meanings.

In terms of affective response, there are two motivational states underlying it, the defensive associated with withdrawal behavior and the appetitive associated with approach behavior (Bradley et al., 2001). As the LPP was observed for both positive and negative stimuli compared with neutral stimuli (Cuthbert et al., 2000; Liu et al., 2012), we cannot decide whether the affective processing elicited by the stadium was approaching or avoidance simply based on the anterior-central LPP effect. Interestingly, the left laterality of LPP in frontal sites suggested that the affective response to the stadium was approaching, as the left laterality of LPP is related to approaching processing (van de Laar et al., 2004; Cunningham et al., 2005; Gable and Harmon-Jones, 2010). Alternatively, as LPP has been suggested to reflect motivated attention to affective stimuli (Keil et al., 2002) and increased motivational significance of affective stimuli (Schupp et al., 2007), the LPP effect may also indicate that stadiums preceded by congruent narratives were able to capture more attentional resources. Moreover, the LPP effect was indifferent to physical differences and the baseline (see Supplementary Material “Analysis of LPP based in another baseline”).

The role of emotion in the relationship between narrative and approach-avoidance response is consistent with the proposal of the SOR model (Mehrabian, 1976). First, previous studies have suggested that narrative was a potent medium to elicit emotion (Westermann et al., 1996). Similarly, the stadium narrative can also serve as an emotional medium and engage participants in emotional processing. Second, although buildings have usually been treated as neutral stimuli, we human beings could establish affective connections with it by narratives, from the perspective of place attachment (Gross and Brown, 2008). This is consistent with the proposition that the stadium could embody emotional connotations like home (Charleston, 2009).

Finally, consistent with previous studies which showed that exposure to narratives shaped our attitudes (Wheeler et al., 1999; Green and Brock, 2000), the behavioral results showed a larger ratio of “enter” after exposure to the impression congruent with the stadium than that of incongruent, indicating that approach-avoidance responses toward the stadium were influenced by the preceding contexts of narratives. The behavioral results confirmed the potent power of a narrative revealed by ERP results. The stadium with a congruent narrative not only elicited larger LPP, which indexed motivational approaching processing, but also induced overt approaching behaviors. The positive correlation between LPP effect and behavioral response once again indicated that the larger the LPP effect, the more likely participants chose to enter the stadium. This suggested that contents of narrative should be selected carefully to make a stadium more appealing for visitors or users; this could be done by highlighting the special features of the stadium purposely. On the other hand, these results further confirmed the interpretation of LPP as motivational approaching processing.

In application, the stadium narrative could serve a multitude of functions for stadium management. On one hand, the stadium could be used as a medium for the expression of specific messages, especially events related to sports, which might be useful for the promotion of the stadium. On the other hand, the narrative provides an effective way for improving the stadium attendance and could be used as a motivator to drive people attending the stadium. Stadium operators could strengthen the affective bonds between the stadium and individuals, and make individuals more attached to the stadium by displaying influential events related to the stadium. Moreover, only one type of building (stadium) was used, the external validity of the study was constrained when applying the results to other buildings. Specifically, when choosing whether or not to enter a stadium, the choice is largely based on preference (Wang et al., 2018), which is not always the case in other buildings. For example, entering a restaurant or hotel is mainly based on necessary needs. We postulate that the influence of a narrative might be different when people chose to enter different buildings, considering that motivation is an important influencing factor in decision-making. Therefore, the specific activities related to the buildings should be considered in real application.

The case study of the stadium also has some implications for buildings. First, the study indicates that buildings are not simply composed of concrete and steel anymore; a narrative is also an important element. A narrative enters the building in many ways; whether it is purely a form or a backstory, it shapes the way we interact with our environment. Therefore, each great building should tell a story to avoid being banal. In management, architectural narrative could be treated as a context to influence the intention of enter or exit, as social context has been shown to affect people’s willingness to buy a house (Guo et al., 2022). For managers, this context could be established by referencing history or by presenting a vision. Second, the same building can convey totally different meanings and affect our perception simply by changing narratives. Reconstruction or renovation of buildings could be achieved by changing narratives, while keeping the original physical structure unchanged. It is the stories between buildings and us and not the silent materials that matters.

## Conclusion and Limitations

To reveal the influence of narratives on an individual’s approach-avoidance attitudes toward the stadium and the underlying neural correlates, we presented a sequence of pictures preceding the reappearance of the stadium and record the EEG simultaneously. Our findings suggested the feasibility of continuity editing procedure to enable a stadium to tell a story. Moreover, both behavioral and electrophysiological results suggest the power of a narrative in shaping our attitudes. The findings provided insights into maximizing the stadium attendance and promotion of the stadium by using narratives. The findings of the case study can also be extended to buildings.

For the convenience of experimental manipulation, we enable the stadium to tell a rather simple story. However, in real life, each great architecture may undergo lots of things and accumulate many stories. Future studies could employ a more vivid story to add interest in the experiment. Moreover, this is an experimental study conducted in a laboratory, which naturally constrained the external validity. In real life, deciding whether or not to enter a building may be subject to many factors and the process of decision-making may be different from that in the laboratory. This could be avoided by using mobile EEG in the future.

## Data Availability Statement

The original contributions presented in the study are included in the article/Supplementary Material, further inquiries can be directed to the corresponding author/s.

## Ethics Statement

The study involving human participants was reviewed and approved by Northwestern Polytechnical University. The patients/participants provided their written informed consent to participate in this study.

## Author Contributions

WZ and QH: conception and study design. HW, XC, YH, YZ, and WZ: data acquisition. WZ, HW, and XC: data analysis. WZ and HW: drafting manuscript. QH, XC, YH, and YZ: revision. All authors contributed to the article and approved the submitted version.

## Conflict of Interest

The authors declare that the research was conducted in the absence of any commercial or financial relationships that could be construed as a potential conflict of interest.

## Publisher’s Note

All claims expressed in this article are solely those of the authors and do not necessarily represent those of their affiliated organizations, or those of the publisher, the editors and the reviewers. Any product that may be evaluated in this article, or claim that may be made by its manufacturer, is not guaranteed or endorsed by the publisher.
