# Supplementary material for: Architectural Narrative Shapes Brain Activities Underlying Approach-Avoidance Response: A Case Study of the Stadium
Source: Front Neurosci. 2022 May 13;16:858888. doi: 10.3389/fnins.2022.858888 (PMC9136322; doi:10.3389/fnins.2022.858888)
Supplement: Supplementary file 1 [file Presentation_1.pdf]

Analysis of LPP based in another baseline

To further confirm the stability of our results. Longer baseline correction was selected (-500, 0). The data analysis were the same as in the formal analysis. Figure S1 shows the grand average waveforms elicited by the fifth picture in the sequence. Figure S2 shows the scalp distribution in the time window of 500–800 ms. Figure S3 shows the grand average waveforms of the congruent-minus-incongruent difference waves derived from the values of fifth picture minus the first picture.

Epoch2 (The fifth picture in the sequence). The results revealed a significant main effect of congruency (midline: $F_{(1,27)}$ =12.66, p = .001, η^2 = .32; lateral: $F_{(1,27)}$ = 11.69, p = .002, η^2 = .30), as the congruent trials elicited a larger LPP than the incongruent trials. Significant interactions between congruency and anteriority were observed on both midline ($F_{(1.31,35.48)}$ = 6.06, p = .01, η^2 = .18) and lateral electrodes ($F_{(1.20,32.41)}$ = 9.12, p = .003, η^2 = .25), as a larger LPP was elicited by the congruent than incongruent trials on anterior electrodes (midline: $F_{(1,27)}$ =11.91, p = .002, η^2 = .31, η_p^2 = .14; lateral: $F_{(1,27)}$ =14.20, p = .001, η^2 = .35) and central electrodes (midline: $F_{(1,27)}$ =15.26, p = .001, η^2 = .36; lateral: $F_{(1,27)}$ =13.37, p = .001, η^2 = .33), but not for the posterior electrodes (midline: $F_{(1,27)}$ =.42, p = .53; lateral: $F_{(1,27)}$ =.64, p = .43).

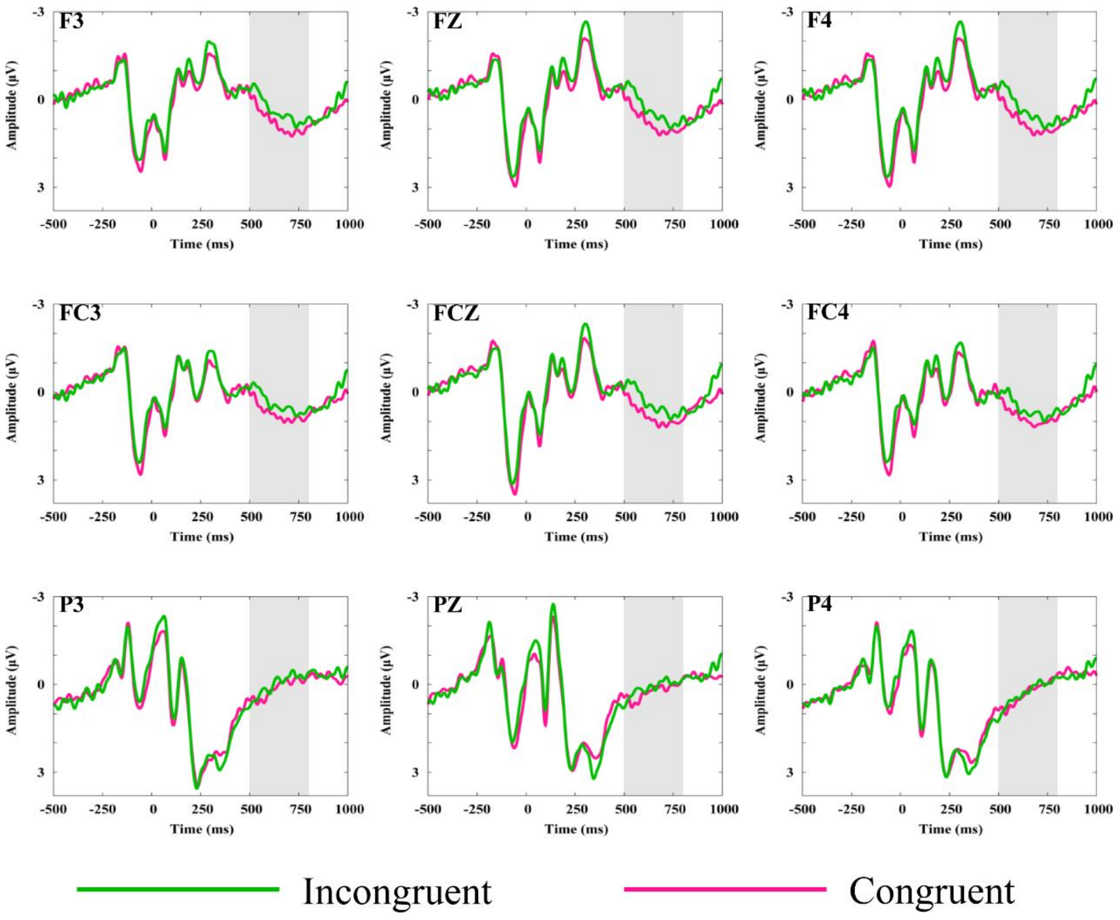

Figure S1. Grand mean ERP waveforms elicited by the reappearance of stadium preceded by congruent or incongruent narratives.

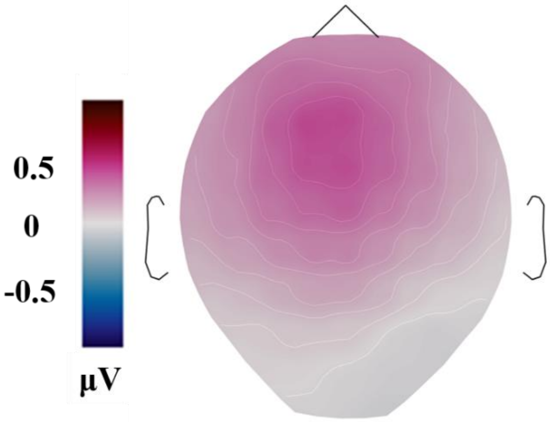

Figure S2. Scalp distribution of the congruent-minus-incongruent difference waves in 500–800 ms.

Laterality of the frontal LPP. The paired t-test showed significant greater difference in the left frontal hemisphere than in the right frontal hemisphere ($t_{(27)} = 4.37$, $p < .001$, $d = 0.22$).

Exclusion of repetition effect

The same picture was presented twice in the present study, which may generate either suppression or enhancement of brain activities and possibly influence the main finding. In order to reveal the relationship between stimulus repetition and modulation of LPP, participants were required to passively view affective or neutral pictures that were repeated 90 times, the results showed decreased LPP amplitude across the habituation (Codispoti, Ferrari & Bradley, 2006). The same pattern was replicated by many studies (Codispoti, Ferrari & Bradley, 2007; Ferrari, 2011; Ferrari, 2015).

In our within-subjects design, the stadium was repeated once in both conditions. Which was not the case as the results showed. Take FZ and FCZ, for example, the amplitude of LPP elicited by the reappearance of stadium did not decreased, but increased (Table S1).

Table S1. Mean value of LPP amplitude for the first and fifth picture in FZ and FCZ

		The first picture	The fifth picture
FZ	congruent	-1.37	0.11
	incongruent	-1.17	-0.21
FCZ	congruent	-1.25	0.04
	incongruent	-0.99	-0.26

In term of epoch 3 (The first picture in the sequence), grand mean ERP waveforms (Figure S3) and corresponding Scalp distribution (Figure S4) were presented.

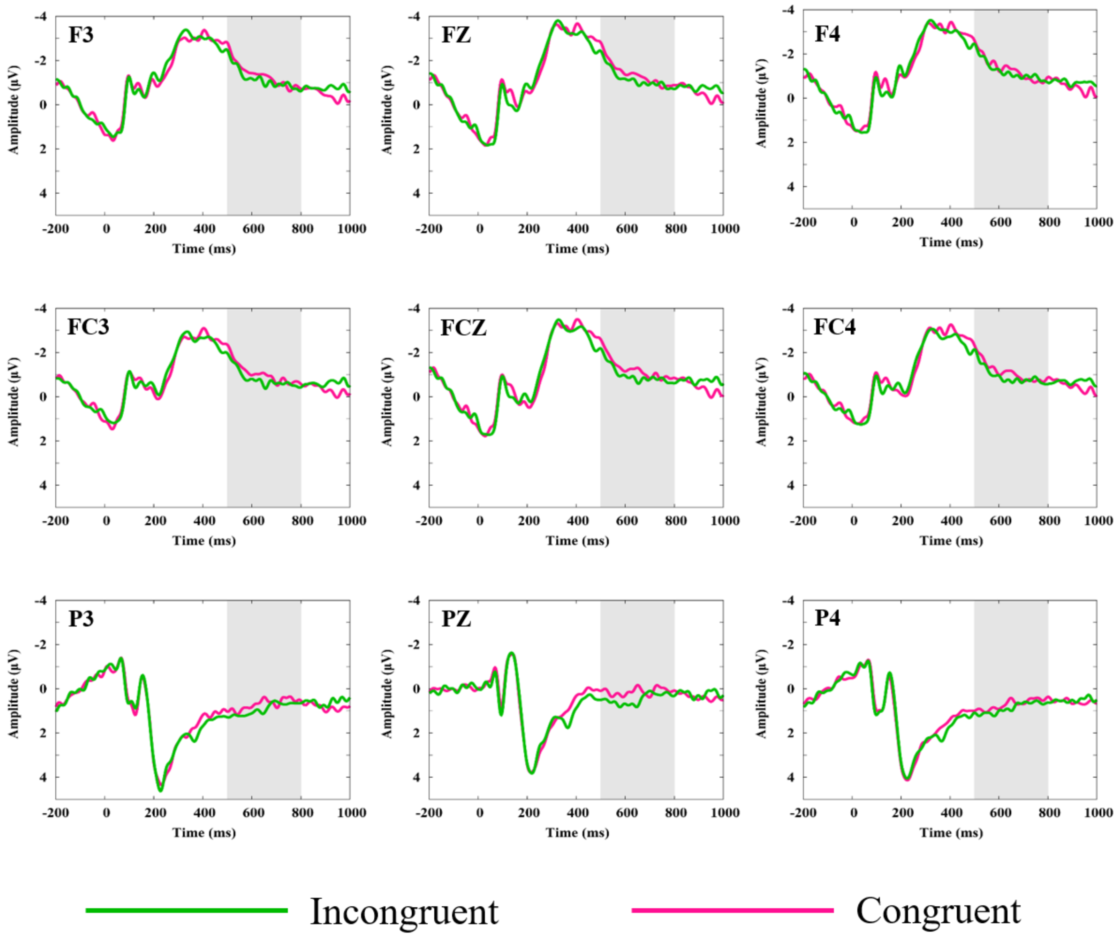

Figure S3. Grand mean ERP waveforms elicited by the first picture.

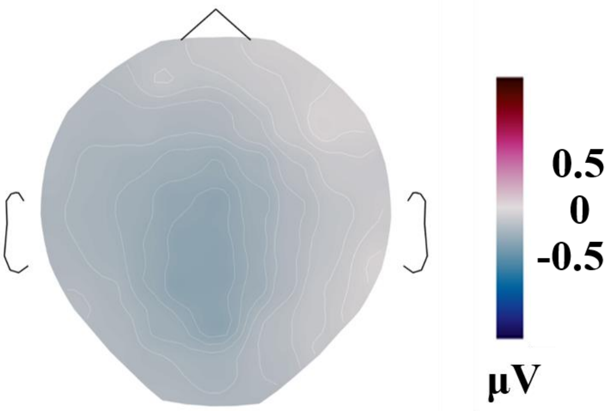

Figure S4. Scalp distribution of the congruent-minus-incongruent difference waves in 500–800 ms for the first picture.

References:

Codispoti, M., Ferrari, V., & Bradley, M. M. (2007). Repetition and event-related potentials: distinguishing early and late processes in affective picture perception. *J Cognitive Neurosci*, *19*(4), 577-586. doi: 10.1162/jocn.2007.19.4.577

Codispoti, M., Ferrari, V., & Bradley, M. M. (2006). Repetitive picture processing: autonomic and cortical correlates. *Brain Res.*, *1068*(1), 213–220. doi:10.1016/j.brainres.2005.11.009

Ferrari, V., Bradley, M. M., Codispoti, M., & Lang, P. J. (2011). Repetitive exposure: brain and reflex measures of emotion and attention. *Psychophysiology*, *48*(4), 515–522. doi: 10.1111/j.1469-8986.2010.01083.x.

Ferrari, V., Bradley, M. M., Codispoti, M., & Lang, P. J. (2015). Massed and distributed repetition of natural scenes: Brain potentials and oscillatory activity. *Psychophysiology*, *52*(7), 865–872. doi: 10.1111/psyp.12424
